# In-vitro inhibitory effect of maternal breastmilk components on rotavirus vaccine replication and association with infant seroconversion to live oral rotavirus vaccine

**DOI:** 10.1371/journal.pone.0240714

**Published:** 2020-11-10

**Authors:** Katayi Mwila Kazimbaya, Caroline C. Chisenga, Michelo Simuyandi, Cynthia Mubanga Phiri, Natasha Makabilo Laban, Samuel Bosomprah, Sallie R. Permar, Sody Munsaka, Roma Chilengi

**Affiliations:** 1 Centre for Infectious Disease Research in Zambia, Lusaka, Zambia; 2 Department of Biomedical Sciences, School of Health Sciences, University of Zambia, Lusaka, Zambia; 3 Department of Biostatistics, School of Public Health, University of Ghana, Legon, Accra, Ghana; 4 Department of Pediatrics, Human Vaccine Institute, Duke University, Durham, North Carolina, United States of America; UAMS/ACHRI/ACNC, UNITED STATES

## Abstract

**Background:**

Despite contributing to a significant reduction in rotavirus associated diarrhoea in highly burdened low- and middle-income countries, live attenuated, oral rotavirus vaccines have lower immunogenicity and efficacy in these settings in comparison to more developed countries. Breastmilk has been implicated among factors contributing to this lowered oral vaccine efficacy. We conducted *in-vitro* experiments to investigate the inhibitory effects of maternal antibody and other non-antibody components in breastmilk on rotavirus vaccine strain (Rotarix) multiplication in MA104 cell culture system and assessed associations with *in-vivo* vaccine seroconversion in vaccinated infants.

**Methods:**

Breastmilk samples were collected from mothers before routine rotavirus vaccination of their infant at 6 weeks of age. For each sample, whole breastmilk, purified IgA, purified IgG and IgG and IgA depleted breastmilk samples were prepared as exposure preparations. A 96 well microtitre plate was set up for each sample including a control in which only MA104 cells were grown as well as a virus control with MA104 cells and virus only. The outcome of interest was 50% inhibition dilution of each of the exposure preparations calculated as the titer at which 50% of virus dilution was achieved. Samples from 30 women were tested and correlated to vaccine seroconversion status of the infant. HIV status was also correlated to antiviral breastmilk proteins.

**Results:**

The mean 50% inhibitory dilution titer when whole breastmilk was added to virus infected MA104 cells was 14.3 (95% CI: 7.1, 22.7). Incubation with purified IgG resulted in a mean 50% inhibitory dilution of 5 (95%CI -1.6, 11.6). Incubating with purified IgA resulted in a mean 50% inhibitory dilution of 6.5 (95% CI -0.7, 13.7) and IgG and IgA depleted breastmilk did not yield any inhibition with a titer of 1.06 (95%CI 0.9, 1.2). Higher milk IgA levels contributed to a failure of infants to seroconvert. HIV was also not associated with any antiviral breastmilk proteins.

**Discussion and conclusion:**

Whole breastmilk and breastmilk purified IgG and IgA fractions showed inhibitory activity against the rotavirus vaccine Rotarix™ whilst IgA and IgG depleted breastmilk with non-antibody breastmilk fraction failed to show any inhibition activity *in-vitro*. These findings suggest that IgA and IgG may have functional inhibitory properties and indicates a possible mechanism of how mothers in rotavirus endemic areas with high titres of IgA and IgG may inhibit viral multiplication in the infant gut and would potentially contribute to the failure of their infants to serocovert. There was not association of HIV with either lactoferrin, lactadherin or tenascin-C concentrations.

## Introduction

Rotavirus vaccines have contributed substantially to mitigate the morbidity and mortality from rotavirus associated diarrhoea [1, 2]. According to reports, rotavirus vaccines have been able to avert approximately 28,000 deaths in under-five children; however, despite these great improvements the reduction in diarrhoea incidence in sub-Saharan Africa and Asia have not been as marked as in other regions [1]. This has been attributed to the reduced rotavirus vaccine efficacy in low and middle-income countries (LMIC) compared to the high-income counterparts [3–5].

There are several reasons postulated to explain the reduced rotavirus vaccine efficacy in LMIC. Among these are maternal immune and non-immune factors; some of which include the antibodies immunoglobulins A (IgA) and G (IgG), human milk olygosaccharides and glycans, mucins and innate components, Lactoferrin (LF), Lactadherin (LA) and Tenascin C (TNC) respectively that are present in breastmilk and acquired by the infant through breastfeeding [6–10]. These breastmilk components have been hypothesized to influence the immunogenicity of the rotavirus vaccines by way of their antiviral neutralizing activity that may inhibit successful replication of the live vaccine virus in the infant gut mucosa and induction of an immune response. Mothers within LMIC are known to have higher exposure to natural rotavirus infection and consequently higher levels of rotavirus specific IgA (RV-IgA) and IgG (RV-IgG) [11, 12]. The transferred antibodies to the infant in breastmilk may potentially bind to and neutralize the live vaccine virus affecting immunogenicity. We have shown in our previous work that infants born to mothers with higher titres of these rotavirus specific antibodies were less likely to seroconvert post vaccination [11]. Lactoferrin(LF) and Lactadherin(LA) are reported to have anti-viral and anti-bacterial activity [13–16] and our previous work demonstrated that increased LA levels were associated with a decrease in the ability of an infant to seroconvert [17]. Other factors thought to impact seroconversion not included here are malnutrition of various micronutrients such as Zinc and Vitamin A, gut microbiome interferences and other disease states such as HIV infection and diarrhoea [18, 19]. Other factors such as rotavirus strain variation have also been thought to contribute to the altered efficacies observed [20, 21].

We investigated the *in-vitro* inhibitory effect of maternal breastmilk (whole breastmilk, purified IgG and IgA, and IgG and IgA depleted breastmilk), collected from breastfeeding mothers of infants receiving rotavirus vaccination in Zambia, against a rotavirus vaccine (Rotarix™) strain. We also described the association of inhibition activity with infant seroconversion to Rotarix™ vaccination. We explored the distribution of breastmilk components by the mother’s HIV status.

## Materials and methods

### Study design

**Fig 1:**
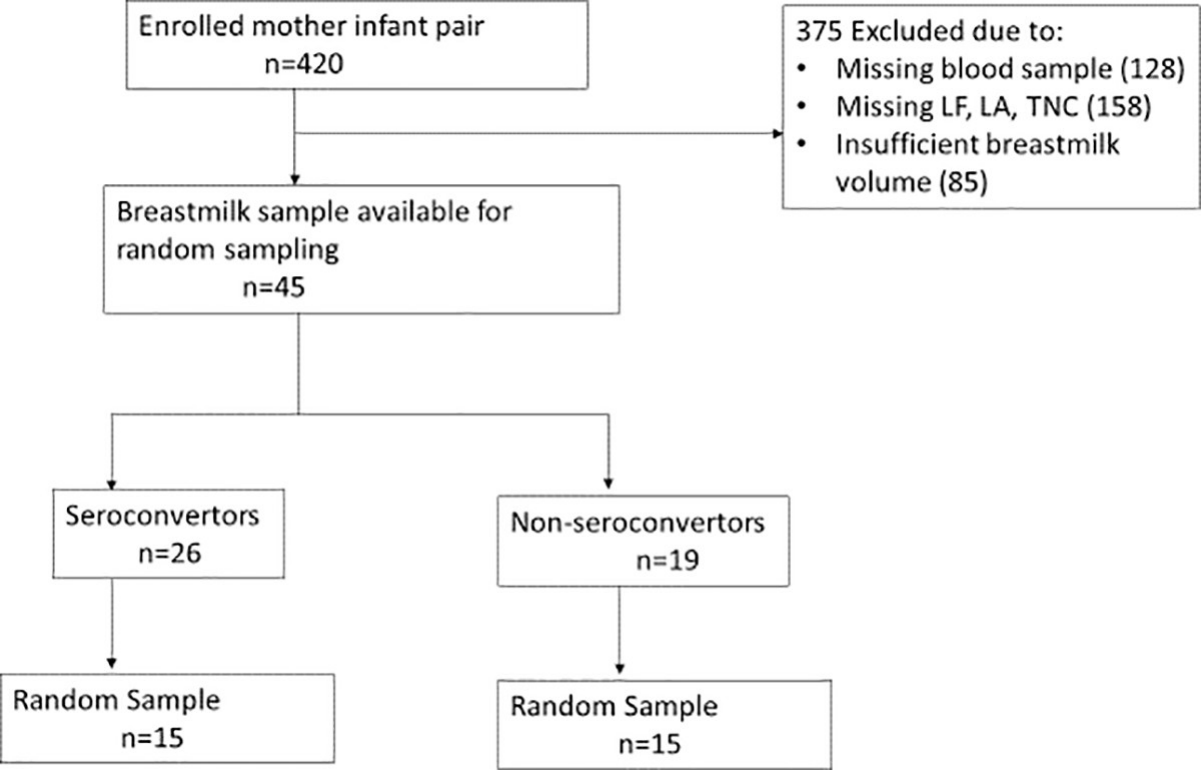
Selection of samples for inhibition assays.

We conducted a laboratory-based exploratory work on different breastmilk components to assess their impact on rotavirus replication in MA104 cells. Freezer stored breastmilk from 420 mother-infant pair cohort recruited from a peri-urban clinic in Lusaka, Zambia in a previously described rotavirus vaccine study between April 2013 and March 2014 [11] were tested for this study. Written consent was obtained from mothers or guardians interested in enrolling their infants in the study after explanation of the study and successful completion of a simple comprehension text. Illiterate mothers were provided information through an impartial witness. The literate impartial witness provided a signed confirmation together with the thumbprint of the participants mother or guardian as confirmed written consent. Breastmilk was collected at baseline before routine first dose Rotarix™ immunization of infant at 6 weeks of age and tested for RV-IgA and RV-IgG titres by enzyme-linked immunosorbent assay (ELISA). The breastmilk was also tested for TNC, LF, and LA levels using standard commercial ELISA kits as previously reported [17]. Following the test, a total of 375 samples were excluded due to missing blood samples (n = 128), and insufficient breastmilk volume (n = 85). A further 158 samples were not tested for LF, LA, TNC, leaving a total of 45 breastmilk samples composed of 26 seroconverters and 19 non-seroconverters from which 15 were selected from each group using a computerised simple random generator (Fig 1). Vaccine seroconversion was defined as ≥ 4 fold rise in infant plasma RV-IgA between baseline and one month following a second dose [11].

### Procedures

#### Separation of breastmilk components and purification

For IgA and IgG purification, breastmilk was thawed to room temperature and split into 1.5ml Eppendorf tubes (Sigma Aldrich Cat No Z606340) and delipidised by centrifugation at 13 000rpm(17 414 g) at 4°C for 5 minutes. The aqueous section of the breastmilk was collected and pooled into 50ml falcon tubes and this was termed as delipidised breastmilk. The pooled fractions were then mixed in a 1:1 ratio of delipidised breastmilk: IgG binding buffer (Pierce, Cat No 21011) and passed through a 1ml slurry suspension (0.5ml bead volume) of Protein G agarose beads (Thermo Scientific Cat No 20399). The flow-through was collected and passed through the beads two more times. The beads were washed with 10ml of IgG binding buffer and IgG was eluted with 5ml Elution Buffer (Thermo Scientific Cat No 21004). 500ul of 1M Tris-HCl was added to the collection tube before elution to neutralize the elution buffer. The procedure was repeated to make two total elutions. The IgG was concentrated with the use of 30K Amicon filters (Merck Cat No UFC803024), collected samples were centrifuged at 4700 rpm (2276 g) for 15 minutes until the complete elution volume passed through the column and buffer exchange was carried out into 1X PBS to a final volume of 500μl. This was stored as concentrated IgG.

**Fig 2:**
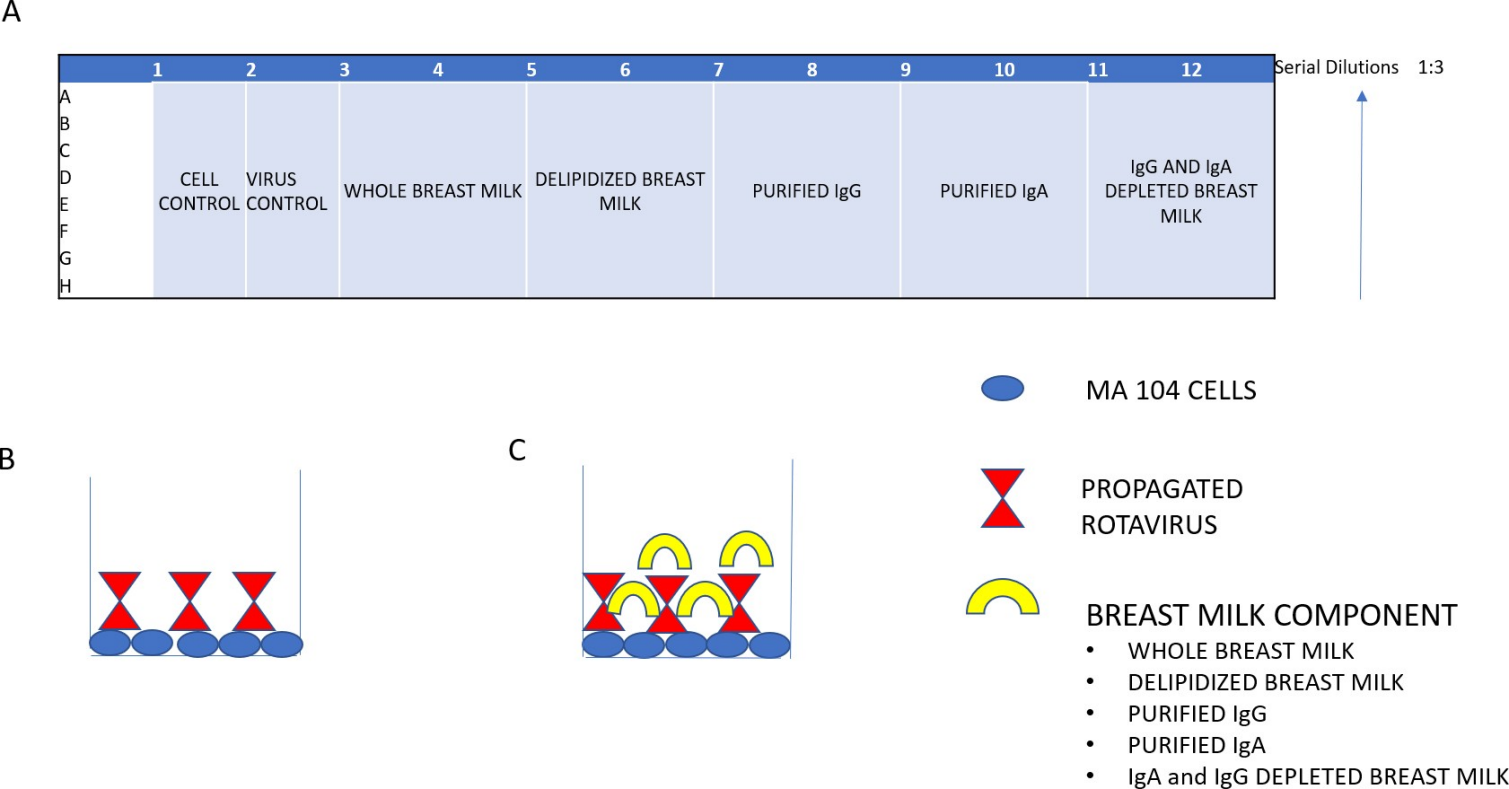
Inhibition plate setup in a 96 well microtitre plate for each breastmilk sample (A). Each plate consisted of a Cell control in which only MA104 cells were grown (Column 1), Virus Control which had MA104 cells and virus only (Column 2, B), the remaining columns constituted MA104 cells, virus and breastmilk sample (columns 5–12, C) (delipidised breastmilk–columns 5 and 6, Purified IgG–columns 7 and 8, purified IgA- columns 9 and 10 and IgA and IgG depleted breastmilk -columns 11 and 12). All breastmilk samples were serially diluted to the ratio of 1:3.

A similar process was completed for IgA purification with Peptide M beads (Invivo Gen Cat No gel-pdm-5). The IgG depleted breastmilk was passed through the beads as before and eluted. Up to 5 elutions were completed to ensure the more abundant IgA was completely eluted. IgA was concentrated with the use of 30K Amicon filters (Merck Cat No UFC803024), collected samples were centrifuged at 4700 rpm (2276 g) for 15 minutes until the complete elution volume passed through the column and buffer exchange was carried out into 1X PBS to a final volume of 500μl. This was stored as concentrated IgA. Fig 2 shows the breast milk purification process as well as the samples stored at each purification stage.

The presence of IgA and IgG and total IgA and IgG concentrations were determined using western blots and Bradfords Assay in the purified IgA and IgG fractions respectively.

#### Inhibition ELISA

The assays were performed as described by Arnold *et al*.,[22]. MA104 cells (Sigma Aldrich Cat No 85102918-1VL) were propagated in 96-well microtitre plates (Thermofisher Cat No 3455) until they were confluent and inoculated with propagated Rotarix (~1.5X10^6^PFU)(GlaxoSmithKline) as well as serially diluted breastmilk for 3 days. The serial dilutions of breastmilk and virus was carried out in a separate plate and incubated for 1 hour prior to addition to the MA104 cells. The plate layout consisted of cell control (column 1) with only MA104 cells, a virus control (column 2) which had MA104 cells and virus only. The sample wells consisted of MA104 cells, virus, as well as breastmilk sample (whole breastmilk (columns 3,4), delipidiszed breastmilk (columns 5,6), purified IgG (columns 7,8) and IgA (columns 9,10) as well as IgA and IgG depleted breastmilk (columns 11,12,) (Fig 2). The plate was then subjected to freeze-thaw cycles and the samples were then tested using standard ELISA (Biotek ELX 808) for Rotavirus antigen presence. 50% inhibition dilution was calculated as the titer at which there was 50% virus as compared to the virus only control (Fig 2). A positive titer indicates a reduction in rotavirus replication, suggesting interference with viral multiplication rate, and thus, “inhibition effect.”

### Sample size calculation

The sample size calculation was based on the primary outcome of mean neutralisation effect. We assumed that the neutralisation effect follows a normal distribution and the true population standard deviation is unknown. We chose a conservative estimate of 20 virus titer for the unknown standard deviation. Therefore, a sample size of 25 is required for a two-sided 95% confidence interval (CI) for the mean neutralisation effect to have the width of no larger than 21 virus titer with a probability of achieving the target CI width of 0.96. The sample size calculation was performed in Stata 16 MP4 (StataCorp, College Station, TX, USA).

### Statistical analysis

The primary outcome was 50% inhibition of viral replication in diffrerent breastmilk fractions. For each of the 30 breastmilk samples included in the analysis, the titer at which there was a 50% reduction (50% inhition dilution (ID50)) in virus titer between the sample and virus control was calculated. A sample was considered as having an inhibition effect if the difference was positive. For our primary analysis, the means of the inhibition for the 30 samples were calculated as a summary measure of inhibition effect. 95% confidence intervals were calculated for the mean. Bar graphs were used to display the ID50 for each of the 30 samples for each breastmilk component. We explored the distribution of breastmilk components LA, LF and TNC by mother’s HIV status using boxplots. Breastmilk specific IgA was categorized into three tiers and IgG was categorized into four tiers. Data was normally distributed and all analyses were performed using Stata 16 (StataCorp, College Station, Texas, USA).

### Ethical statement

The study was approved by the University of Zambia Biomedical Research Ethics Committee (Protocol #011-09-15), the University of North Carolina at Chapel Hill Institutional Review Board and the Zambian National Health Research Authority (Protocol #101/23/10/1). The study was conducted following the principles of the Declaration of Helsinki and compliance with good clinical practice guidelines; ClinicalTrials.gov registration number NCT 01886833. Breastmilk samples were collected following informed consent and Good Clinical Practice (GCP) guidelines.

## Results

### Characteristics of participants

The primary study enrolled 420 mother-infant pairs. After previous studies screened for LA, LF, TNC, infant seroconversion data and volume of breastmilk sample, a total of 45 (26 seroconverters and 19 non-seroconverters) had adequate samples available for this study, from which 15 seroconverters and 15 non seroconverters (30 samples) were randomly selected for analysis. About two-thirds (20/30, 67%) of mothers were HIV-positive (Table 1). Approximately half of the mothers were in the lowest tier for breastmilk rotavirus-specific IgA (56.7%) and IgG levels (43.3%) (Table 1).

**Table 1:** 50% Inhibitory dilution (ID50) Mean breastmilk samples titers (Whole breastmilk (WBM), purified IgG, purified IgA, and IgA and IgG depleted breastmilk) by mother’s characteristics.

	# (% of total)	WBM		IgG		IgA		IgA and IgG Depleted	
**HIV Status**		**Mean (SD)**	**95% CI**	**Mean (SD)**	**95% CI**	**Mean (SD)**	**95% CI**	**Mean (SD)**	**95% CI**
Negative	10 (33.3)	18.3 (28.6)	-2.2, 38.8	1 (0)	1, 1	3.3 (7.2)	-1.9, 8.5	1 (0)	1, 1
Positive	20 (66.7)	13.2 (16.4)	5.5, 21.0	7 (21.6)	-3.1, 17.1	8.1(23.1)	-2.7, 19.0	1.1 (0.5)	0.9, 1.3
**Breastmilk Rotavirus Specific IgA Titer (tertiles)**									
1 “1–80”	17 (56.7)	20.5 (25.7)	7.3, 33.8	6.6 (23.0)	-5.3, 18.4	6.5(23.0)	-5.3, 18.4	1(0)	1,1
2 “160”	6 (20)	6.0 (9.0)	-3.5, 15.5	1 (0)	1,1	4.8 (9.4)	-5.0, 14.7	1 (0)	1, 1
3 “320”	7 (23.3)	8.9 (8.1)	1.3, 16.4	4.6 (8.6)	-3.4, 12.5	7.7 (17.8)	-8.7, 24.1	1.2 (0.76)	06, 2.0
**Breastmilk Rotavirus Specific IgG Titer (quartiles)**									
1 “320–2560”	13 (43.3)	17.2 (26.9)	0.9, 33.4	1 (0)	1,1	8.3 (26.3)	-7.6, 24.2	1 (0)	1,1
2 “5120”	5 (16.7)	15.4 (20.6)	-10.2, 41.0	20 (42.4)	-32.7, 72.8	1 (0)	1,1	1 (0)	1,1
3 “10240”	7(23.3)	13.4(15.9)	-1.3, 28.1	4.3 (8.6)	-3.7, 12.3	7.7(17.8)	-8.7, 24.1	1(0)	1,1
4 “20480”	5 (16.7)	10.8(12.3)	-4.5, 26.0	1.4 (0.8)	0.29, 2.5	5.6(10.3)	-7.2, 18.4	1.4 (0.9)	0.3, 2.5
**Total**	**30 (100)**	**14.3(20.9)**	**7.1, 22.7**	**5 (17.6)**	**-1.6, 11.6**	**6.5(19.3)**	**-0.7, 13.7**	**1.06 (0.4)**	**0.9,1.2**

### Inhibition ability of breastmilk components

**Fig 3:**
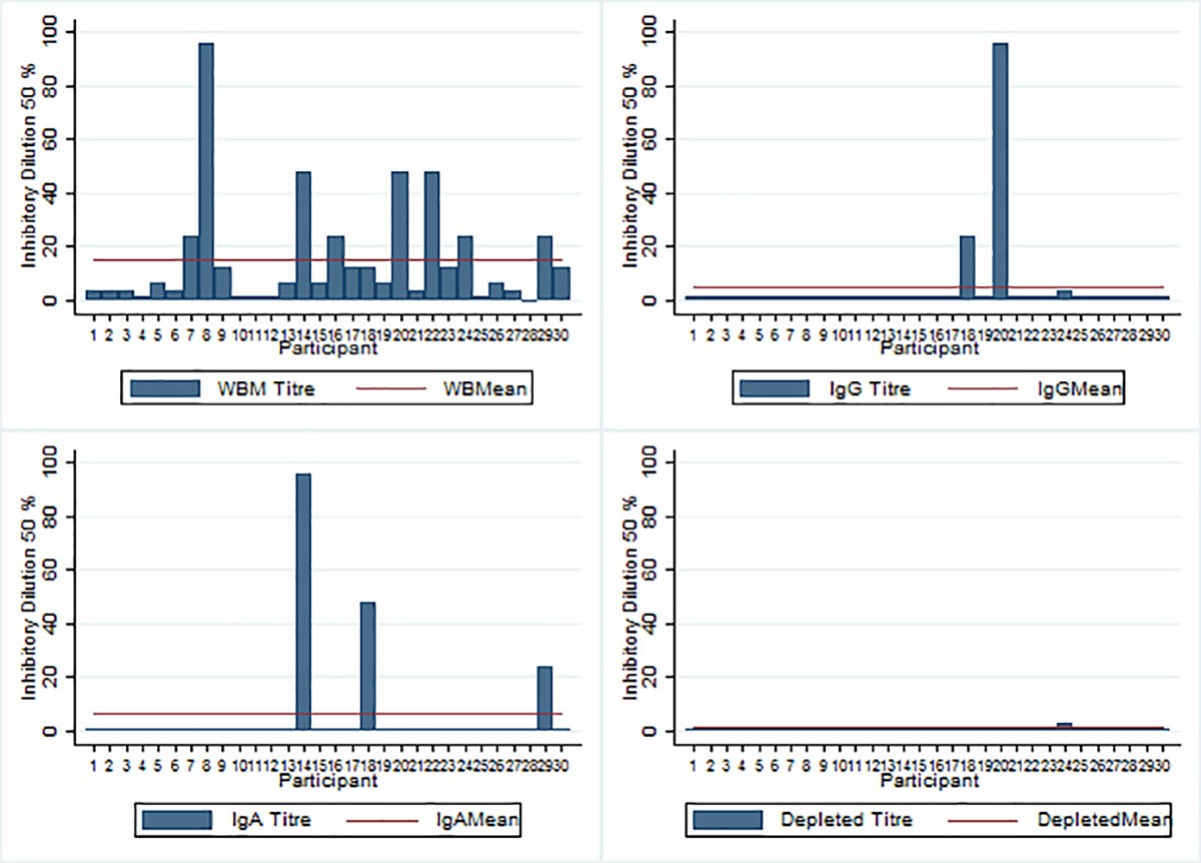
50% Inhibition dilution titer by individual sample for each breastmilk samples (Whole breastmilk, purified IgG, purified IgA, and IgA and IgG depleted breastmilk) that inhibited viral replication by 50% compared to virus only control. The red horizontal line shows the mean 50% inhibition dilution titer.

When whole breastmilk was added to virus prior to infection of MA104 cells, the mean 50% inhibition dilution titer was 14.3 (95% CI: 7.1, 22.7) (Table 1). Likewise, incubation of the virus with purified IgG and IgA gave a mean 50% inhibition dilution titer of 5 (95%CI -1.6, 11.6) and 6.5 (95% CI -0.7, 13.7) respectively (Table 1). Incubating the virus with IgG and IgA depleted breastmilk did not yield any inhibition as they had a mean ID50 breastmilk titer of 1.06 (95%CI 0.9, 1.2) (Table 1). About 87% (26/30) of the samples demonstrated inhibition by whole breastmilk whereas less than 10% demonstrated any inhibition by purified IgG and IgA and one by IgA and IgG depleted breastmilk (Fig 3).

### Association of breastmilk IgA with infant seroconversion

**Fig 4:**
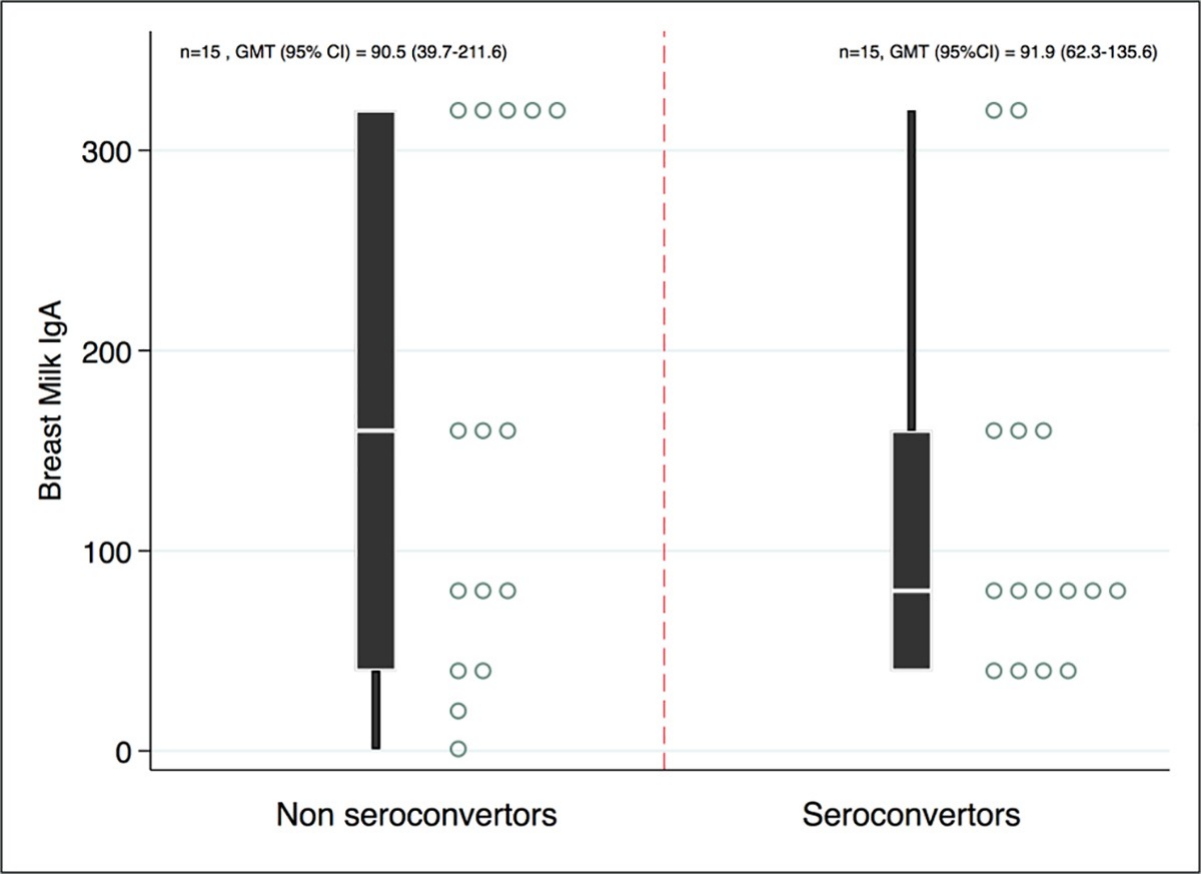
Distribution of rotavirus specific breastmilk IgA titers by seroconversion status.

Although not statistically significant, rotavirus-specific IgA levels in breastmilk showed an inverse correlation with infant vaccine seroconversion, suggesting a trend of failure to serovconvert with higher maternal milk rotavirus-specific IgA titers (Fig 4).

### Breastmilk components (LA, LF and TNC) by HIV status

**Fig 5:**
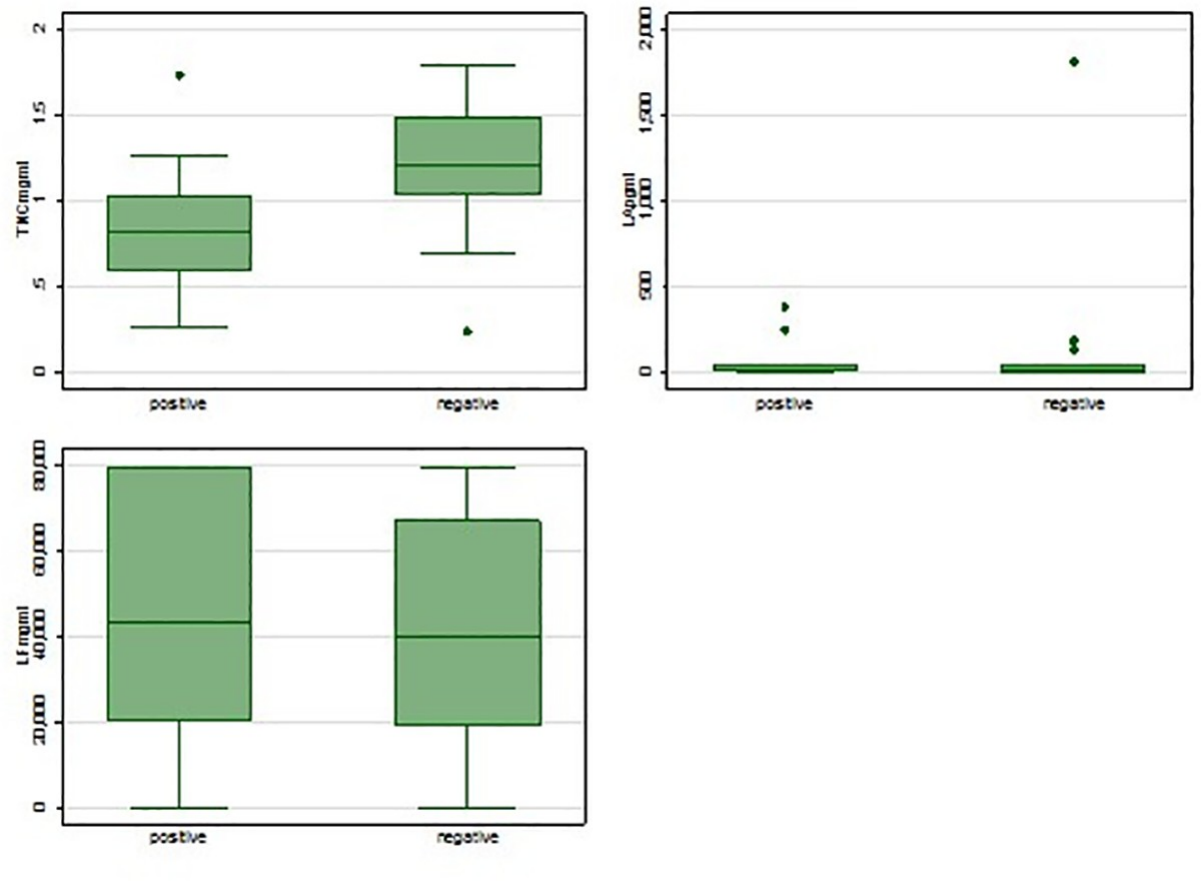
Distribution of breastmilk components concentration (lactoferrin, lactadherin and tenascin-C) by mother’s HIV status.

The distribution of LF and LA was generally similar between HIV-infected and uninfected mothers whereas that of Tenascin-C was generally elevated among HIV-uninfected mothers (Fig 5). As IgA and IgG depleted breastmilk was used as a proxy for the combined effect of these three proteins (LF, LA and TNC) or any other breastmilk components as no inhibition was observed in that breastmilk fraction.

## Discussion

This exploratory work aimed at showing how different breastmilk components would impact rotavirus vaccine replication in MA104 cells as a proxy to gut infection and viral replication. Presuming that such viral replication is an important prerequisite for antigenic stimulation in the gut that results in vaccine IgA seroconversion that is considered an important indication of vaccine efficacy. To the best of our knowledge, this is the first effort attempting to replicate *in-vivo* activity in the GI tract by *in-vitro* experiments to validate or refute the hypothesised mechanisms of effects of maternal pre-existing anti-rotavirus immunity on vaccine efficacy.

Studies elsewhere have investigated the effect of breastmilk on rotavirus vaccine efficacy. Some research has compared vaccine-elicited responses of breast-feeding to bottle-feeding infants, assessed withholding breastmilk at the time of vaccination and investigated the effect of these on infant seroconversion. However, this research has shown no statistically significant difference in seroconversion between bottle and breast-fed infants, although formula-fed infants were more likely to seroconvert [23–25]. Other research studies have shown that withholding breastmilk at the time of vaccination does not affect the ability of the child to seroconvert after rotavirus vaccination [26–28], although this is inconclusive for several reasons including the length of time that an infant can ethically be restricted from breastfeeding [29].

While whole breastmilk showed inhibition of vaccine virus, the lower purified milk IgG potency against vaccine virus compared to IgA (5 95%CI -1.6, 11.6) indicate that IgG is less potent than IgA for vaccine inhibition. Although the full functional activity of IgG is also not fully understood, some studies found that mothers rotavirus-specific IgG levels in milk could be considered a potential inverse correlate of protection [19, 30–32]. Few samples of purified IgG here had any detectable inhibition. Total IgG is notably lower in breastmilk than IgA [33] which may account for the lower levels observed here for ID50 and rotavirus specific IgG could also have been lower in concentration in the purified fractions than in whole breastmilk as the IgA and IgG depleted samples were not tested for total rotavirus specific IgG and IgA concentration. It was also observed that whole breastmilk had a higher titer than both purified IgA and IgG, so while some of this may be accounted for by the fact some of the IgA and IgG may not have been rotavirus specific it may also point to the fact that there are other breastmilk components not included in this study that may have contributed to this. Human milk oligosaccharides (HMOs) are synthesized glycosyltransferases also considered to be prebiotic agents encouraging growth of beneficial microorganisms [10]. HMOs in various conjugate forms are also recognized as soluble decoy receptors that will bind pathogens that have an affinity for oligosaccharides that are expressed in the infants gut [10]. Rotavirus infection may also be mitigated in this way and may also explain some of the observations in this study. Mucins and glycosaminoglycans are also known to block infection by viruses and bacteria and could also be attributed to these observations [10, 34]. Another plausible reason for the lack of viral replication observed could be that the removal of the lipid layer may have removed some other important anti-viral components that work in conjunction with IgG and IgA.

Most published work on rotavirus immunology and breastmilk has concentrated on the immunological aspects [12, 35–37]; but this research has not only attempted to generate *in-vitro* mechanistic evidence, we have also tried to explain the actual observed vaccine seroconversion in Zambian infants. The real strength in this work is that we utilized breastmilk samples obtained from mothers whose infants were immunized and have triangulated the clinical infant vaccine seroconversion data with the maternal breast milk viral inhibition experiments.

While we previously found an association between failure to seroconvert and exposure to high levels of LA in maternal breastmilk [17], these experiments have not shown an inhibitory effect of this moiety, and neither the others we explored (LF and TNC). The antimicrobial effects of TNC have also been reported against HIV [38, 39], but we have not seen any effect on rotavirus replication in MA104 cells. There may be several reasons for this: firstly, we undertook the experience after storing the samples for several years and the freeze-thaw cycles could have affected the molecular integrity of the breastmilk components, additionally the samples were not delipidized prior to freezing, therefore there may be small fat globules in the purified samples that could affect the results; secondly, we only evaluated 30 samples in which we observed large confidence intervals suggesting that our study may not have been sufficiently powered to detect a small effect, and much less any differences between HIV exposed and non-exposed samples. Third, the inability to test for TNC, LA and LF in the IgA and IgG depleted samples due to the concentrations below the limit of detection (LOD) of the kits is another major limitation. The final limitation here is that the purified IgA and IgG was not assessed for rotavirus specificity, which would have put greater context on some of the observed results.

The limitations alluded to earlier could be overcome by specifically design studies with well-considered sample sizes and assays on fresh samples.

The failure to show any effect by LF on inhibition is unexpected, however there may be other justifiable reasons for this. The various functions of the proteins could be another reason for the failure to see any inhibitory activity. For example, LFs key role has been demonstrated to sequester iron and inhibit bacterial growth, therefore despite its presence in breastmilk its main target function could be iron sequestering which is not involved in viral replication and thus allow minimal protein for other activities in the breastmilk [40–42]. A similar theory could be applied to TNC.

The results on IgA and IgG-depleted breastmilk is quite compelling to us. An ID50 of 1.06 (95%CI 0.9, 1.2) suggests an absolute non-inhibiiton in the absence of IgG and IgA. These data suggest that IgA and IgG may have functional inhibitory properties which have been suggested by others [30, 35, 43], and were previously also observed *in-vivo* [11]. The data here alludes to mothers in rotavirus endemic areas having high titres of whole breastmilk IgA and IgG and these can possibly inhibit viral multiplication in the infant gut, similar to the effect in the MA104 cell culture system [43–45]. These results hold some potentially controversial interpretation; that breastmilk in disease-endemic areas has a negative effect on rotavirus vaccine effectiveness. Unfortunately, these are the same environments where malnutrition would pose a much greater and more immediate threat; as such withholding breastmilk is not be a reasonable policy option. More research studies and better understanding are required here. Parenteral routes would completely overcome this problem, and vaccines in development such as the novel non-replicating sub-unit P2-VP8 vaccine under development by PATH offers great hope [46]. Indeed this approach has been successfully utilized in the polio field where the oral vaccine is now being replaced by an injectable one [47].

## Conclusion

Our study is a substantial contribution as it opens possibilities into detailed research to confirm or refute hypotheses on mechanism of action in the limiations of rotavirus vaccine efficacy and its relationship to maternal immunity. It also affirms the current advocacy to switch to parenteral vaccines in order to ensure that low and middle income countries can overcome some of the factors affecting vaccine efficacy and see improvements in rotavirus related morbidity and mortality.
